# Pathogenicity of missense variants affecting the collagen IV α5 carboxy non-collagenous domain in X-linked Alport syndrome

**DOI:** 10.1038/s41598-022-14928-x

**Published:** 2022-07-04

**Authors:** Joel T. Gibson, Omid Sadeghi-Alavijeh, Daniel P. Gale, Hansjörg Rothe, Genomics England Research Consortium, Omid Sadeghi-Alavijeh, Daniel P. Gale, Judy Savige, J. C. Ambrose, P. Arumugam, E. L. Baple, M. Bleda, F. Boardman-Pretty, J. M. Boissiere, C. R. Boustred, H. Brittain, M. J. Caulfield, G. C. Chan, C. E. H. Craig, L. C. Daugherty, A. de Burca, A. Devereau, G. Elgar, R. E. Foulger, T. Fowler, P. Furió-Tarí, A. Giess, J. M. Hackett, D. Halai, A. Hamblin, S. Henderson, J. E. Holman, T. J. P. Hubbard, K. Ibáñez, R. Jackson, L. J. Jones, D. Kasperaviciute, M. Kayikci, A. Kousathanas, L. Lahnstein, K. Lawson, S. E. A. Leigh, I. U. S. Leong, F. J. Lopez, F. Maleady-Crowe, J. Mason, E. M. McDonagh, L. Moutsianas, M. Mueller, N. Murugaesu, A. C. Need, C. A. Odhams, A. Orioli, C. Patch, D. Perez-Gil, M. B. Pereira, D. Polychronopoulos, J. Pullinger, T. Rahim, A. Rendon, P. Riesgo-Ferreiro, T. Rogers, M. Ryten, K. Savage, K. Sawant, R. H. Scott, A. Siddiq, A. Sieghart, D. Smedley, K. R. Smith, S. C. Smith, A. Sosinsky, W. Spooner, H. E. Stevens, A. Stuckey, R. Sultana, M. Tanguy, E. R. A. Thomas, S. R. Thompson, C. Tregidgo, A. Tucci, E. Walsh, S. A. Watters, M. J. Welland, E. Williams, K. Witkowska, S. M. Wood, M. Zarowiecki, Judy Savige

**Affiliations:** 1 Department of Medicine (Melbourne Health and Northern Health), The University of Melbourne, Parkville, VIC 3050 Australia; 2 Department of Renal Medicine, University College London, London, UK; 3 Genomics England, Queen Mary University of London, London, UK; 4 Centre for Nephrology and Metabolic Disorders, 02943 Weisswasser, Germany; 5 Genomics England, London, UK; 6 William Harvey Research Institute, Queen Mary University of London, London, EC1M 6BQ UK

**Keywords:** Genetics, Nephrology

## Abstract

X-linked Alport syndrome is a genetic kidney disease caused by pathogenic *COL4A5* variants, but little is known of the consequences of missense variants affecting the NC1 domain of the corresponding collagen IV α5 chain. This study examined these variants in a normal (gnomAD) and other databases (LOVD, Clin Var and 100,000 Genomes Project) to determine their pathogenicity and clinical significance. Males with Cys substitutions in the collagen IV α5 NC1 domain reported in LOVD (n = 25) were examined for typical Alport features, including age at kidney failure. All NC1 variants in LOVD (n = 86) were then assessed for structural damage using an online computational tool, Missense3D. Variants in the ClinVar, gnomAD and 100,000 Genomes Project databases were also examined for structural effects. Predicted damage associated with NC1 substitutions was then correlated with the level of conservation of the affected residues. Cys substitutions in males were associated with the typical features of X-linked Alport syndrome, with a median age at kidney failure of 31 years. NC1 substitutions predicted to cause structural damage were overrepresented in LOVD (*p* < 0.001), and those affecting Cys residues or ‘buried’ Gly residues were more common than expected (both *p* < 0.001). Most NC1 substitutions in gnomAD (88%) were predicted to be structurally-neutral. Substitutions affecting conserved residues resulted in more structural damage than those affecting non-conserved residues (*p* < 0.001). Many pathogenic missense variants affecting the collagen IV α5 NC1 domain have their effect through molecular structural damage and 3D modelling is a useful tool in their assessment.

## Introduction

Alport syndrome is the commonest genetic kidney disease and results from pathogenic variants in *COL4A3*, *COL4A4* or *COL4A5*^1,2^. These genes encode the collagen IV α3, α4 and α5 chains that trimerise to form the collagenous network found in the basement membranes of the kidney, ear and eye^3^.

X-linked Alport syndrome results from pathogenic variants in *COL4A5* and is the cause of severe disease. Males typically develop kidney failure before the age of 30, together with hearing loss and ocular abnormalities^4,5^. Females have a more variable phenotype and are usually less severely affected, with up to 20% developing kidney failure by the age of 60^6,7^. Autosomal recessive Alport syndrome results from pathogenic variants in both copies of *COL4A3* or *COL4A4*^2,8^, resulting in a phenotype similar to X-linked disease in males^9^. Autosomal dominant Alport syndrome, formerly known as ‘thin basement membrane nephropathy’, results from heterozygous pathogenic variants in *COL4A3* or *COL4A4*^10^. Most affected individuals have only haematuria, some develop late-onset kidney failure, but hearing loss and ocular features are very rare^11,12^.

Each collagen IV α-chain comprises three domains: the non-collagenous 7S domain at the amino terminus, the intermediate collagenous (Gly-X–Y) domain, and the non-collagenous NC1 domain at the carboxy terminus^3^. The mature collagen IV α-chains, unlike the fibril-forming collagens, retain the two non-collagenous termini, which have important structural and functional roles^13^. The NC1 domains direct chain stoichiometry and trimerisation^14,15^, and interactions between the termini on neighbouring trimers are responsible for network formation within the basement membranes^16^. Post-translational modifications and ligand-binding sites have also been identified within the non-collagenous domains^3,17^.

The six collagen IV NC1 domains are highly conserved. Each comprises two homologous subdomains and is stabilised through six intrachain disulphide bonds^16^. During trimer formation, the three NC1 domains interact through a domain swapping mechanism where a β-hairpin motif from each chain is exchanged with a docking region (VR3) on the adjacent chain^13,14^. Following trimerisation, the NC1 domains of two identical trimers interact end-to-end to form a hexamer, which is stabilised by six covalent sulphilimine cross-links and extensive hydrophobic and hydrophilic interactions^16,18–20^. These hexamers have an important structural function within the collagenous network, and are a focal point of bioactivity within the glomerular basement membrane^21^.

Missense variants affecting the NC1 domains are reported increasingly, but their pathogenicity and clinical significance are often difficult to determine. In the collagen IV α5 chain, many of these variants have been associated with later onset kidney failure in affected males, but the phenotypes and extrarenal features vary^22–25^. Cys substitutions are the commonest type, probably because of the structural importance of disulphide bonds for folding of the NC1 domain. The 12 NC1 Cys residues are conserved in all six collagen IV α-chains, and considered ‘critical domains’^26^. Little is known of other mechanisms underlying disease caused by NC1 missense variants, but conformational changes that interfere with trimer assembly or function are likely to be important^15,20^.

This study determined the clinical and molecular implications of missense variants affecting the NC1 domain of the collagen IV α5 chain. Firstly, the expected phenotype for Cys substitutions was established and compared with previously-published data for collagenous Gly substitutions in the same molecule^27^. A structural modelling tool was then used to identify additional variants that resulted in further damaging change. Finally, the importance of conserved residues in the NC1 domain was examined.

## Methods

### Reference sequences

*COL4A5* variants were described at the genomic and protein levels using the following NCBI reference sequences: NM_033380.3, NP_203699.1. Isoform 2 was used, since this is the predominant isoform expressed in the kidney. The NC1 domain was defined as beginning at the first residue immediately following the final Gly-X–Y of the collagenous domain, consistent with the literature and ending at the final residue before the stop codon (Supplemental Table 1). This is different from Uniprot which defines the NC1 domain as beginning at the first secondary structure (beta-strand) following the collagenous domain, meaning that 5 or so residues in each chain are not included in the collagenous or NC1 domain. A further difference is that Uniprot reports positions based on isoform 1.

### Variant databases

Variants were primarily examined using the Leiden Open Variation Database (LOVD; https://www.lovd.nl)^28^. This is an open source database comprising variants, often with associated demographic and clinical data from mainly individuals with X-linked Alport syndrome, which have been extracted from the literature or submitted directly by collaborating laboratories. It now includes more than 2,090 unique *COL4A5* variants. Variant pathogenicity was assessed by the submitting laboratory, or where this was not provided, using the American College of Medical Genetics (ACMG) criteria as assessed by VarSome (https://varsome.com).

Three further datasets that included *COL4A5* variants were used for comparative analysis with LOVD:

- Genomics England 100,000 Genomes Project (100kGP)—this database comprises hospital records and genomic data from individuals and families with various diseases, including some with X-linked Alport syndrome or other genetic kidney diseases. Genomic data were extracted from the aggregate multi-sample VCF (aggV2), comprising 78,195 germline genomes from the 100kGP (http://genomicsengland.co.uk; version 10 data release)^29^.
- ClinVar—this database contains variants, phenotypes and pathogenicity assessments submitted by testing laboratories, and includes more than 550 Pathogenic or Likely Pathogenic *COL4A5* variants (https://www.ncbi.nlm.nih.gov/clinvar; accessed 21 November 2021).
- Genome Aggregation Database (gnomAD)—this database includes exomic and genomic data from cases and controls recruited for various studies of cardiac, psychiatric and other diseases, which have been combined to represent a cross-section of the general population. Individuals have not been selected for kidney disease, and the database is not expected to be enriched for rare pathogenic *COL4A5* variants (https://gnomad.broadinstitute.org; v2.1.1; accessed 15 November 2021)^30^.

### IRB approval

Use of the 100kGP data and publication of this manuscript were approved by the Genomics England Research Ethics Committee. This study did not require Institutional Review Board permission because all other datasets (LOVD, ClinVar, and gnomAD) were de-identified and already in the public domain. All variants in these datasets were from individuals who, at recruitment, had provided written informed consent to testing, the submission of variants to the database, and the use of these variants in research. Testing and submission in each case was performed with Institutional Review Board approval and according to the principles of the Declaration of Helsinki. LOVD also included clinical data associated with variants where this was found in the primary published manuscripts, where Institutional Review Board approval was provided in each case.

### Computational tools

#### Predicted splicing changes

Exonic nucleotide substitutions in *COL4A5* close to canonical splice sites sometimes affect normal splicing, especially when the final nucleotide of an exon is affected^31–33^. To ensure that variants with unreported splicing effects were not included unintentionally, all variants within 3 bases of a canonical splice site were assessed using MaxEntScan^34^. Mutant and wild type sequences were scored using the maximum entropy model, and variants were considered likely to affect splicing where the mutant score was at least 15% lower than the wild type score^35^. Variants predicted to affect normal splicing were then excluded from the subsequent analyses (Supplemental Table 2).

#### Predicted structural changes

Structurally-damaging variants were identified using Missense3D (http://missense3d.bc.ic.ac.uk)^36,37^. This tool uses three-dimensional structural information from experimentally-determined protein models to predict the consequences of amino acid substitutions. Sixteen different structural features were examined (buried charge introduced, buried charge replaced, buried charge switch, etc.), but the ‘cis Pro replaced’ feature was not included in this study because there are no cis Pro residues in the collagen IV α5 NC1 domain. Variants were analysed using the experimentally-determined structure of the collagen IV α5 chain (UniProt ID: P29400, PDB code: 5NAZ).

The sensitivity and specificity of this tool were examined using the data in ClinVar, where the damage prediction for each missense variant affecting the collagen IV α5 NC1 was compared with the reported pathogenicity. Variants of uncertain significance (VUS) and variants with conflicting interpretations of pathogenicity were excluded. Forty-three unique NC1 missense variants were identified in ClinVar, of which 11 were reported as ‘Pathogenic’ or ‘Likely Pathogenic’, and 8 as ‘Benign’ or ‘Likely Benign’. Missense3D correctly classified 15 of the 19 (79%) variants, with a sensitivity of 0.64 and specificity of 1.00.

### Cys substitutions

Individuals with *COL4A5* variants resulting in Cys substitutions in the NC1 domain were identified in LOVD. Clinical data were extracted for all affected males, and survival analysis performed to determine the median age at kidney failure. This was compared with the median age at kidney failure in males with non-Cys substitutions in the NC1 domain identified in LOVD, and with previously-published data for males with collagen IV α5 Gly substitutions in the collagenous domain^27^.

Age at kidney failure was defined as the age at clinical diagnosis of kidney failure, or where this was not available, the age at commencing dialysis, or at the first kidney transplant. Each family was included once only. Where multiple males from the same family developed kidney failure, the mean age (or median age, where only this was available) at kidney failure was used. Where a range of ages was reported, the midpoint was used. Where an affected male had not yet developed kidney failure, his age at the most recent review was included as a censored data point. Males with multiple pathogenic variants in *COL4A3*-*COL4A5*, or families with only affected females were not included.

Cys substitutions reported in the ClinVar, gnomAD and 100kGP databases were also examined.

### Other structurally-damaging variants

Predicted structural changes were obtained for missense variants affecting the NC1 domain reported in LOVD. The observed proportion of variants with each structural change was then compared with the theoretical expected frequencies to determine whether any changes were overrepresented.

The expected proportions of variants with each structural change were calculated using a neighbour-dependent nucleotide substitution rate model^38^. This model was chosen since it takes into account the higher rate of substitutions observed for transitions than transversions, as well as the effect of adjacent nucleotides. In brief, all theoretically-possible nucleotide substitutions resulting in missense variants affecting the collagen IV α5 NC1 domain were computed and assessed for structural damage (Supplemental Fig. 1). The relative frequency of each variant was then determined using the relative substitution rates set out by the model, and the total expected proportion of variants causing each structural change calculated.

This analysis was repeated for all collagen IV α5 missense variants affecting the NC1 domain found in 100kGP and gnomAD. 

### Variants affecting conserved residues

The final part of this study examined whether substitutions affecting conserved residues in the collagen IV α5 NC1 domain were more likely to result in structural damage than those affecting non-conserved residues. This analysis used the cohort of all theoretically possible collagen IV α5 NC1 missense variants described above, but only included unique amino acid substitutions for each residue without regard to the underlying DNA change (Supplemental Fig. 1).

The level of conservation for each residue was determined from multiple sequence alignment of the reference sequences for the six human collagen IV α-chains (Supplemental Table 1) using Clustal Omega (https://www.ebi.ac.uk/Tools/msa/clustalo/). Residues were ‘conserved’ where the same amino acid was present in each of the 6 chains.

Finally, variants affecting conserved residues in LOVD were examined in isolation, and the comparison of observed and expected frequencies of damaging variants performed above was repeated. The expected frequencies were recalculated for this purpose, considering only conserved residues. Variants affecting conserved residues in gnomAD were also examined separately.

### Statistical analysis

Categorical data were represented in contingency tables and compared using Fisher’s exact test. Observed and expected frequencies were compared using the exact binomial test.

A *p*-value less than 0.05 was considered significant, and a Bonferroni correction accounting for 15 tests was applied for comparisons of the individual structural changes. All statistical analysis was performed in R (version 3.6.2) and used the *survival* and *survminer* packages^39–41^. Survival curves were produced using the Kaplan–Meier method and compared using the log-rank test.

### Significance statement

X-linked Alport syndrome is a genetic kidney disease resulting from pathogenic *COL4A5* variants. Missense changes affecting the NC1 domain of the corresponding collagen IV α5 chain are identified increasingly, but their pathogenicity and clinical significance are often uncertain. This study established the expected phenotype for Cys substitutions (the most common NC1 variant), and identified additional likely pathogenic variants using a new 3D structural modelling tool. Predicted structurally-damaging variants were more likely to affect conserved residues and were overrepresented in a pathogenic variant database. Cys substitutions were associated with the typical features of Alport syndrome, and most males with NC1 variants developed later-onset kidney failure. 3D structural modelling represents a useful tool for assessing the pathogenicity of variants in the NC1 domain.

## Results

### Cys substitutions

Twenty-nine unique Cys substitutions in 49 families were identified in LOVD (Table 1). These variants affected all 12 Cys residues in the collagen IV α5 NC1 domain, with substitutions resulting in all 6 possible amino acids.

**Table 1:** Collagen IV α5 Cys residues and missense variants reported in LOVD, ClinVar and gnomAD.

Residue	Conserved^a^	LOVD	ClinVar	gnomAD^b^
Cys1482	Yes	Cys1482Gly (n = 2), LP Cys1482Phe (n = 1), P	Cys1482Phe (n = 1), P Cys1482Trp (n = 1), P	
Cys1515	Yes	Cys1515Phe (n = 1), P	Cys1515Tyr (n = 1), VUS	
Cys1527	Yes	Cys1527Ser (n = 2), LP		Cys1527Ser (n = 1)
Cys1533	Yes	Cys1533Arg (n = 1), LP Cys1533Phe (n = 1), LP		
Cys1570	Yes	Cys1570Arg (n = 1), P Cys1570Ser (n = 1), P Cys1570Tyr (n = 3), LP	Cys1570Arg (n = 1), P Cys1570Phe (n = 1), LP Cys1570Ser (n = 4), P	
Cys1573	Yes	Cys1573Arg (n = 4), P Cys1573Ser (n = 1), P Cys1573Tyr (n = 1), LP	Cys1573Arg (n = 1), P Cys1573Ser (n = 1), P	
Cys1592	Yes	Cys1592Arg (n = 2), P/LP Cys1592Phe (n = 2), VUS/P Cys1592Tyr (n = 2), LP	Cys1592Arg (n = 1), P Cys1592Phe (n = 1), P	
Cys1626	Yes	Cys1626Ser (n = 2), LP/P	Cys1626Ser (n = 1), VUS	
Cys1638	Yes	Cys1638Arg (n = 1), P Cys1638Ser (n = 1), P Cys1638Trp (n = 1), LP Cys1638Tyr (n = 4), P/LP	Cys1638Gly (n = 1), P Cys1638Trp (n = 1), LP Cys1638Tyr (n = 1), P	
Cys1644	Yes	Cys1644Ser (n = 1), LP Cys1644Trp (n = 1), LP Cys1644Tyr (n = 3), LP	Cys1644Arg (n = 1), VUS Cys1644Ser (n = 1), P Cys1644Tyr (n = 3), P	
Cys1684	Yes	Cys1684Arg (n = 1), P Cys1684Trp (n = 1), P Cys1684Tyr (n = 4), P/LP	Cys1684Arg (n = 1), P Cys1684Trp (n = 1), P	
Cys1687	Yes	Cys1687Gly (n = 1), LP Cys1687Phe (n = 1), P Cys1687Tyr (n = 2), P	Cys1687Gly (n = 1), P Cys1687Phe (n = 1), P	

No Cys missense variants were found in the 100kGP database. LB, Likely Benign; LP, Likely Pathogenic; P, Pathogenic; VUS, Variant of Uncertain Significance.

^a^Residues fully conserved in all six human collagen IV α-chains.

^b^Independent pathogenicity assessments were not provided by this database.

**Figure 1:**
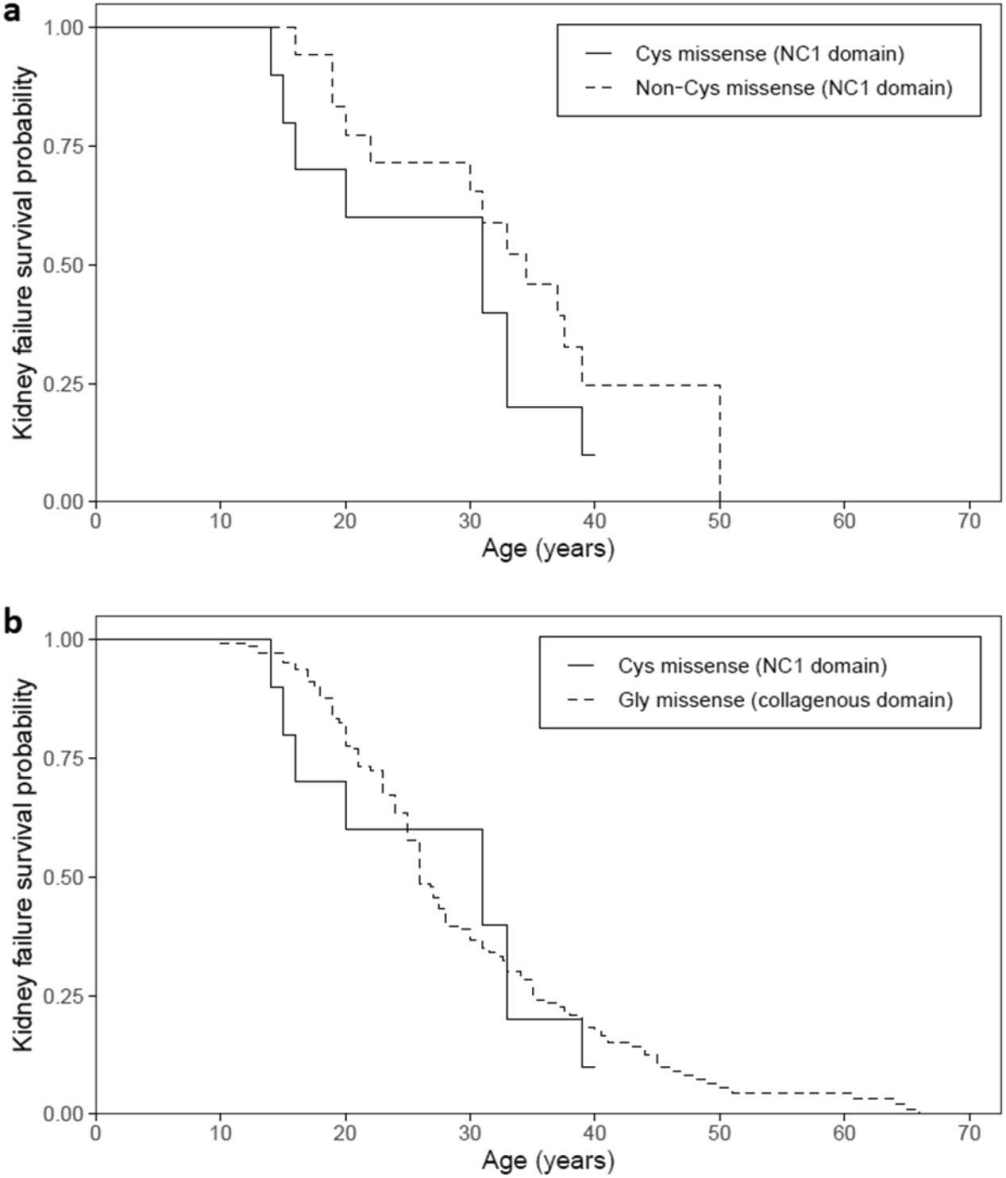
Proportion of cases without kidney failure for males with collagen IV α5 missense variants reported in LOVD. Cys missense variants in the NC1 domain were compared with **(a)** other missense variants in the NC1 domain (*p* = 0.23) and **(b)** Gly missense variants in the Gly-X–Y collagenous domain (*p* = 0.77). Censored data points are not shown.

Cys substitutions were present in 25 families each with at least one affected male, 7 families with only affected females, and 17 families where the sex of the affected individuals was not reported. In the families with affected males, 13 (of the 18 with clinical data, 72%) had at least one male with kidney failure. The median age at kidney failure was 31 years (n = 13) (Table 2). This was not different from the median age at kidney failure for males with other non-Cys substitutions in the NC1 domain (34.5 years, n = 22, *p* = 0.23), or males with Gly substitutions in the collagenous domain (26 years, n = 157, *p* = 0.77) (Table 2, Fig. 1). Eleven of these families (11/15, 73%) had at least one male with a hearing loss, and 3 (3/8, 38%) with ocular changes.

**Table 2:** Median ages at kidney failure for males with collagen IV α5 missense variants reported in LOVD.

	N with kidney failure	Median age at kidney failure (years) (IQR)	*p*-value^a^
Cys missense (NC1 domain) (n = 13)	9 (69%)	31 (16, 33)	–
Non-Cys missense (NC1 domain) (n = 22)	13 (59%)	34.5 (22, 39)	0.23
Gly missense (collagenous domain) (n = 157)^b^	129 (82%)	26 (21, 35)	0.77

*IQR* Interquartile range.

^a^Comparison with Cys missense (NC1 domain).

^b^Data previously reported^27^.

Only one Cys substitution (p.Cys1527Ser) was found in gnomAD (Table 1). This was reported in a heterozygous female. The same variant was reported in LOVD in a 40 year old male with proteinuria, stage 3 chronic kidney disease and hearing loss, and also in a female (no age recorded) with haematuria and renal insufficiency.

Twenty-one unique Cys substitutions were found in ClinVar, affecting 10 of the 12 Cys residues (Table 1). Sixteen of these substitutions were also present in LOVD.

No Cys substitution was identified in the 100kGP database.

### Other structurally-damaging variants

**Figure 2:**
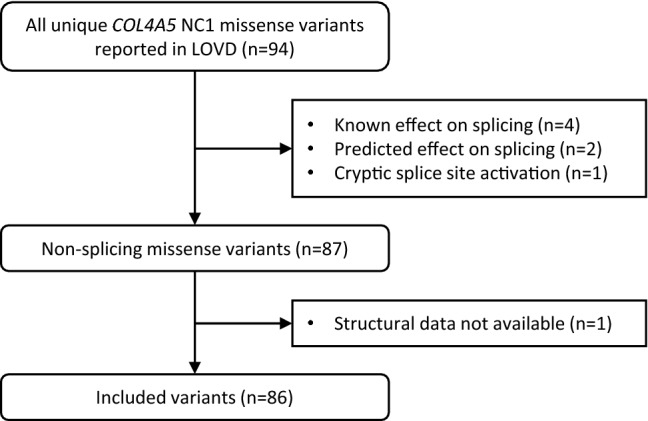
Variant inclusion flowchart for the LOVD study cohort. Counts refer to the number of unique missense variants.

**Figure 3:**
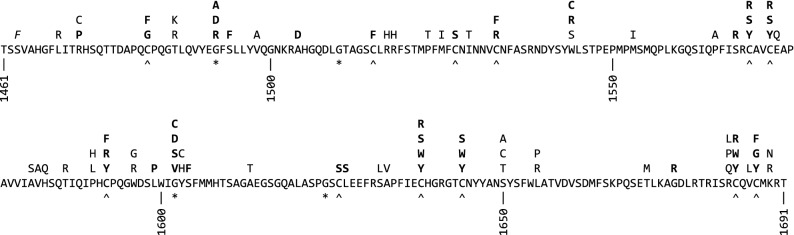
Amino acid sequence of the collagen IV α5 NC1 domain. Substitutions found in LOVD are indicated above the sequence. Substitutions predicted to cause structural damage are in bold, and substitutions where no structural data was available are in italics. Cys residues are indicated with a caret (^), and buried Gly residues with an asterisk (*).

Eighty-six unique missense variants affecting the NC1 domain were found in LOVD and included in this analysis (Figs. 2, 3). Forty-six (53%) were predicted to cause a structurally-damaging change, which was greater than the expected frequency of 19% (*p* < 0.001) (Table 3a). Three specific structural changes were found more often than expected. These were ‘disulphide breakage’, ‘clash’ and ‘buried Gly replaced’ (all *p* < 0.001).

**Table 3:** Predicted structural changes caused by collagen IV α5 NC1 missense variants reported in LOVD.

	a. All substitutions (n = 86)			b. Excluding Cys substitutions (n = 57)		
	Observed	Expected (%)	*p*-value^a^	Observed	Expected (%)	*p*-value^a^
Any structural damage	46 (53.5%)	19.2	** < 0.001**	17 (29.8%)	14.5	**0.004**
Structural change						
Buried charge introduced	8 (9.3%)	3.4	0.01	4 (7.0%)	2.9	0.08
Buried charge replaced	0	1.2	0.63	0	1.3	1
Buried charge switch	0	0.5	1	0	0.5	1
Buried Gly replaced	7 (8.1%)	1.2	** < 0.001**	7 (12.3%)	1.3	** < 0.001**
Buried H-bond breakage	4 (4.7%)	4.5	0.80	4 (7.0%)	4.8	0.35
Buried hydrophilic introduced	5 (5.8%)	1.6	0.01	1 (1.8%)	0.9	0.40
Buried Pro introduced	0	1.3	0.63	0	1.4	1
Buried salt bridge breakage	0	1.0	1	0	1.1	1
Buried/exposed switch	2 (2.3%)	1.7	0.66	2 (3.5%)	1.6	0.23
Cavity altered	1 (1.2%)	0.6	0.39	1 (1.8%)	0.6	0.29
Clash	14 (16.3%)	2.7	** < 0.001**	3 (5.3%)	1.0	0.02
Disallowed phi/psi	3 (3.5%)	3.0	0.75	3 (5.3%)	3.2	0.43
Disulphide breakage	29 (33.7%)	5.5	** < 0.001**	–	–	–
Gly in a bend	0	0.4	1	0	0.4	1
Secondary structure altered	1 (1.2%)	1.0	0.60	1 (1.8%)	1.1	0.47

Significant values are in bold.

^a^For comparisons of each individual structural change the significance threshold was set at *p* < 0.0033, accounting for 15 tests.

Disulphide breakage from Cys substitutions is a known disease-causing mechanism, and as such, Cys substitutions are reported by testing laboratories as Likely Pathogenic. Interestingly, even after Cys substitutions were excluded to ensure that they did not bias the results, predicted structurally-damaging variants were still found more often than expected in LOVD (*p* = 0.004) (Table 3b). However, ‘buried Gly replaced’ was the only feature that remained overrepresented in this database (*p* < 0.001).

Forty unique NC1 missense variants were found in the 100kGP database. Of the 38 variants with corresponding structural data, only 5 (13%) were predicted to be damaging. This was not different from the expected frequency of 19% (*p* = 0.42). These variants were found in 6 heterozygous females, including one with haematuria, and another with haematuria and proteinuria, but none had evidence of impaired renal function. No male with a predicted structurally-damaging NC1 missense variant was identified in this database.

Seventy-one unique NC1 missense variants were reported in gnomAD. Only 8 of 68 (12%) variants with available structural data were predicted to be structurally-damaging, which was again not different from the expected frequency of 19% (*p* = 0.16). Most (6/8, 75%) structurally-damaging variants were found once only, and none was found more than 3 times.

#### Buried Gly variants

The collagen IV α5 NC1 domain includes 4 buried Gly residues, each of which is conserved in all six human collagen IV α-chains (Table 4). Buried Gly residues were identified using Missense3D. The α5 chain also includes 13 non-buried Gly residues, but none of the variants affecting these residues in LOVD or ClinVar was reported as pathogenic.

**Table 4:** Collagen IV α5 Gly residues and missense variants reported in LOVD, ClinVar, 100kGP and gnomAD.

Residue	Conserved^a^	LOVD	ClinVar	100kGP^b^	gnomAD^b^
**Buried Gly residues**					
Gly1492	Yes	Gly1492Arg (n = 3), P/LP Gly1492Ala (n = 5), P/LP Gly1492Asp (n = 3), P	Gly1492Val (n = 2), LP Gly1492Ala (n = 2), VUS Gly1492Asp (n = 1), P		
Gly1510	Yes		Gly1510Arg (n = 1), P^c^		
Gly1602	Yes	Gly1602Cys (n = 2), P/LP Gly1602Ser (n = 2), P/LP Gly1602Val (n = 4), P/LP Gly1602Asp (n = 2), P	Gly1602Ser (n = 1), LP Gly1602Val (n = 1), P Gly1602Asp (n = 1), P		
Gly1624	Yes			Gly1624Cys (n = 1)	Gly1624Ala (n = 1)
**Non-buried Gly residues**					
Gly1467	Yes			Gly1467Ala (n = 1)	
Gly1485	Yes				
Gly1500	Yes				
Gly1506	No		Gly1506Ser (n = 1), LB	Gly1506Cys (n = 1) Gly1506Ser (n = 1)	Gly1506Cys (n = 3) Gly1506Ser (n = 2) Gly1506Val (n = 1)
Gly1513	Yes				
Gly1560	No		Gly1560Asp (n = 1), VUS		
Gly1595	No	(Gly1595Val (n = 1), P)^d^	(Gly1595Val (n = 1), P)^d^		
Gly1612	Yes				
Gly1615	Yes				
Gly1617	Yes				
Gly1640	Yes				
Gly1642	Yes				
Gly1675	No	Gly1675Arg (n = 1), VUS			

*LB* Likely Benign, *LP* Likely Pathogenic, *P* Pathogenic, *VUS* Variant of Uncertain Significance.

^a^Residues fully conserved in all six human collagen IV α-chains.

^b^Independent pathogenicity assessments were not provided by these databases.

^c^c.4528G > C is predicted to affect normal splicing, so may not cause the expected p.Gly1510Arg missense change (Supplemental Table 2).

^d^c.4784G > T has been demonstrated to activate a cryptic splice site rather than cause the expected p.Gly1595Val missense change^44^.

**Figure 4:**
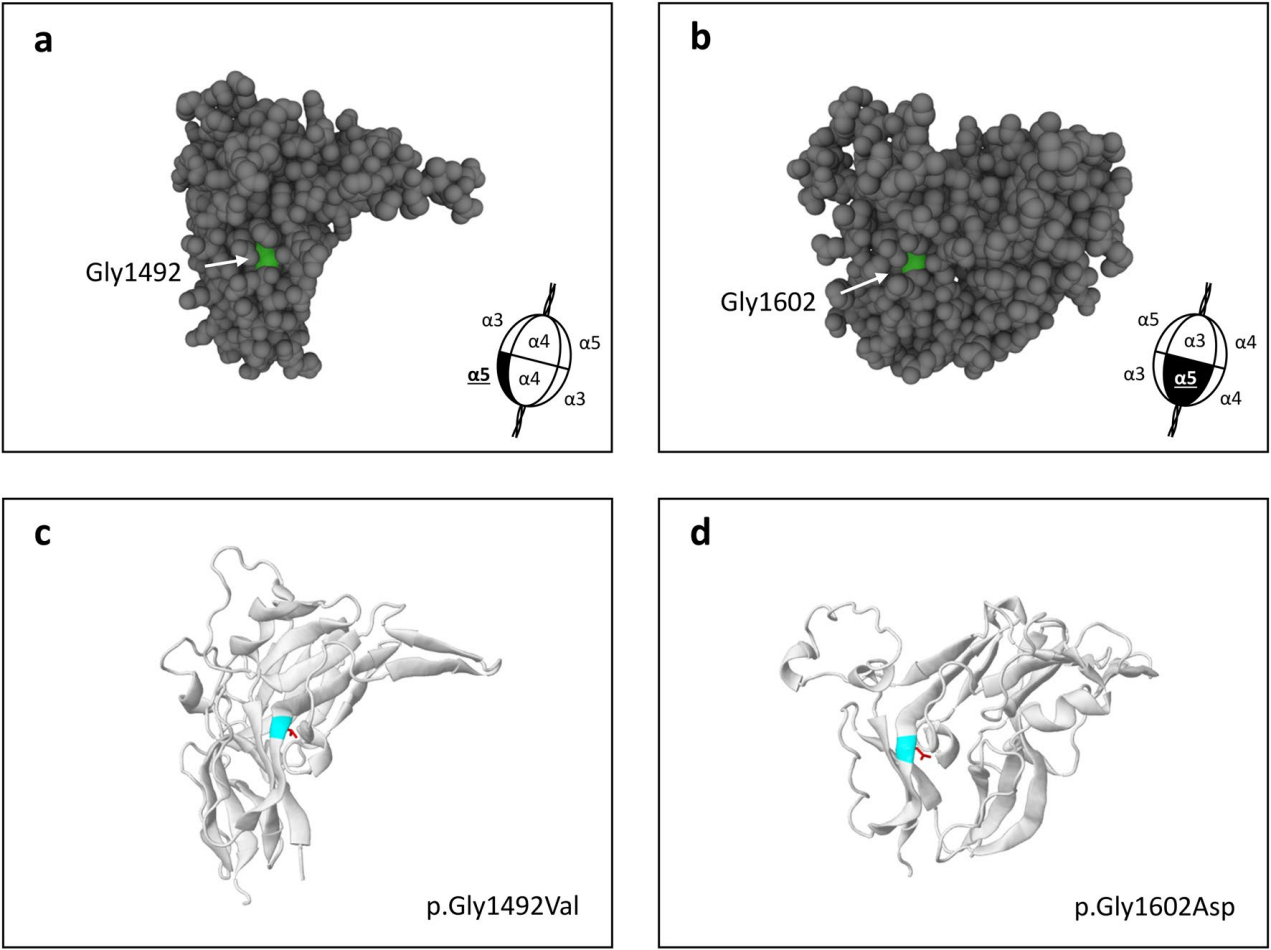
**(a,b)** Space-filling models showing the location of the two commonly affected buried Gly residues (shown in green) within the collagen IV α5 NC1 domain. Models were produced using UniProt (https://www.uniprot.org/). **(c,d)** Ribbon diagrams showing the effect of two substitutions affecting these buried Gly residues (shown in blue). In both cases the affected residue is located within a β-strand, where the larger side-chain of the substituting residue (shown in red) encroaches upon the neighbouring secondary structure. Diagrams were produced using JSmol, accessed through Missense3D (http://missense3d.bc.ic.ac.uk).

Seven unique missense variants affecting buried Gly residues were found in 21 families in LOVD, and all involved Gly1492 or Gly1602 (Fig. 4). They included 10 families with at least one affected male, 6 families with only affected females, and 4 families where the sex of the affected individual was not reported. One additional male demonstrated a mosaic pattern of expression for a buried Gly variant and was excluded.

Clinical data were rarely reported for these variants. Of the few males with clinical data, only 1 of 6 (17%) had kidney failure. This was less often than described above for Cys substitutions (*p* = 0.05). Four of 5 (80%) males also had a hearing loss, but none of three had ocular abnormalities.

Many of the same buried Gly variants found in LOVD were also reported in ClinVar (Table 4). Interestingly, ClinVar also included a pathogenic variant affecting the Gly1510 residue (p.Gly1510Arg), which was likely to affect splicing (Supplemental Table 2).

Only one buried Gly variant (p.Gly1624Cys) was found in the 100kGP database. This was in a heterozygous female with kidney stones and unexplained hearing loss, but no evidence of impaired kidney function. A different variant affecting the same residue (p.Gly1624Ala) was found in gnomAD, and also in a heterozygous female. This was the only buried Gly variant found in gnomAD. Neither variant was present in LOVD or ClinVar.

### Variants affecting conserved residues

The collagen IV α5 NC1 domain comprises 231 residues, 105 (45%) of which are fully conserved in all six human collagen IV α-chains (Supplemental Fig. 2). Considering all possible exonic single-nucleotide substitutions, 1,345 unique missense variants are possible, of which structural data were available for 1,316 (Supplemental Fig. 1). Variants affecting conserved residues were more likely to result in a structurally-damaging change compared with variants affecting non-conserved residues (*p* < 0.001) (Supplemental Table 3a). This association persisted even after excluding all Cys variants (*p* < 0.001) (Supplemental Table 3b).

Sixty-three (63/86, 73%) unique collagen IV α5 NC1 missense variants in LOVD affected a conserved residue. To determine whether the conservation status of a residue could be used as a proxy for whether a variant affecting that residue were structurally-damaging, these 63 variants were examined in isolation. The expected frequencies of structurally-damaging variants were also recalculated using only conserved residues. Interestingly, even when considering only conserved residues, predicted structurally-damaging variants were still found more often in LOVD (44/63, 70%) than the new expected frequency of 32% (*p* < 0.001). This increase persisted even after excluding all Cys variants (*p* = 0.006). In contrast, of the 22 unique collagen IV α5 NC1 missense variants affecting a conserved residue in gnomAD, only 4 (18%) were predicted to cause structural damage. This was a lower proportion than found in LOVD (*p* < 0.001). These results suggest that structural modelling provides useful information about a variant’s likely pathogenicity that the level of conservation alone does not fully capture.

## Discussion

In X-linked Alport syndrome, missense variants affecting the NC1 domain of the collagen IV α5 chain are recognised increasingly but little has been known of their pathogenicity or clinical consequences. This study used a new computational tool to predict the structurally-damaging changes associated with missense variants reported in four variant databases.

Cys residues in the NC1 domain of the collagen IV α5 chain are highly conserved and have been recognised recently as ‘critical domains’ in assessing pathogenicity^26^. Variants resulting in Cys substitutions were the most common pathogenic change in the NC1 domain and rare in the general population. In the collagen IV α5 chain, substitutions have been reported for all 12 NC1 Cys residues and were associated with all the typical clinical features of Alport syndrome. The median age at kidney failure for males was more than 30 years, but not different from that for other NC1 substitutions, nor for collagenous domain Gly substitutions^27. ^ However, the median age at kidney failure for both NC1 groups suggest that variants in this region cause later-onset disease, and the lack of a difference with the Gly substitutions may have been due to the small cohort.

Missense variants predicted to cause structural damage to the NC1 domain were overrepresented in LOVD, suggesting that this was a major cause of pathogenicity. Three types of damage were more common than expected: disulphide breakages, clashes, and substitutions of buried Gly residues. Interestingly, many Cys substitutions flagged the ‘clash’ feature in addition to the expected ‘disulphide breakage’, possibly because disulphide bonds necessarily bring two segments of the chain into close proximity. Thus clashes were not more common after Cys variants had been excluded. In contrast, only a small number of variants in gnomAD were predicted to cause structural damage, consistent with structurally-neutral variants being more common in the general population. Interestingly, about 12% of the NC1 variants in gnomAD were predicted to be structurally-damaging, which was similar to the frequency of structurally-damaging variants reported for non-disease associated variants in the original Missense3D study (11%)^36^.

Missense variants affecting a buried Gly residue were common in LOVD, and all involved Gly1492 or Gly1602. These two residues are located in analogous positions in the two homologous NC1 subdomains, so that they may have an important structural role. However, only one of the affected males had kidney failure, suggesting that they may be associated with milder disease. Gly substitutions affecting the Gly-X–Y collagenous domain are the most common missense variants reported in Alport syndrome^26^. These Gly residues are located within the core of the triple helix, where their small size is essential for close packing of the chains^42^. Substitution with any other amino acid destabilises heterotrimer formation, resulting in disease^43^. Buried Gly variants in the NC1 domain may have their effect through a similar mechanism, where substitutions with larger amino acids destabilise the molecule or impair folding of the NC1 domain.

Missense variants affecting conserved residues were more likely to result in structural damage than those affecting non-conserved residues. This suggests an important structural function for conserved residues in the NC1 domain of the α5 chain, and considering the homology between α-chains similar findings are to be expected for the α3 and α4 chains. Interestingly, a greater proportion of variants affecting conserved residues in LOVD were predicted to be structurally-damaging than those affecting conserved residues in gnomAD. This highlights the importance of using structural data in addition to conservation status when assessing a variant’s likely pathogenicity. Residues that are fully conserved in all six α-chains are likely to be critical for the NC1 domain structure. However, many chain-specific residues may also be important for functions such as chain recognition during trimerization^13,14^.

Many in silico pathogenicity prediction tools return only a binary outcome for variants such as ‘benign' or ‘deleterious'. While this is useful for identifying likely pathogenicity it provides little explanation of the mechanism by which the amino acid substitution affects protein structure. Missense3D addresses this issue by examining sixteen specific structural changes that have previously been associated with disease-causing variants^36^. Using this tool we have identified buried Gly residues in the NC1 domain that represent novel mutational ‘hotspots’ in Alport syndrome. Missense3D also allows for the analysis of both experimentally-determined protein structures as well as predicted structures. This means that Missense3D represents a valuable addition to the tools available for variant assessment.

This study examined the molecular and clinical implications of missense variants affecting the NC1 domain of the collagen IV α5 chain. It characterised the expected phenotype of Cys substitutions, evaluated a new computational tool for assessing pathogenicity, and identified additional likely pathogenic variants. However, variant classification in the NC1 domain remains challenging, and other molecular factors such as the effect on ligand binding sites may also be important^17^. Further studies will confirm whether the present study's findings are also applicable to NC1 variants in the collagen IV α3 and α4 chains, and the implications for autosomal dominant and recessive Alport syndrome. Structural modelling is one of many tools now available for assessing variants in at least X-linked Alport syndrome.

## Supplementary Information

Supplementary Information.

## Data Availability

The public datasets analysed in the present study are freely available in the LOVD (https://databases.lovd.nl/shared/genes/COL4A5), ClinVar (https://www.ncbi.nlm.nih.gov/clinvar/), and gnomAD (https://gnomad.broadinstitute.org/) repositories. Data from the 100,000 Genomes Project is available to authorised users for approved projects through the Genomics England Research Environment (http://genomicsengland.co.uk).
