# Supplementary material for: Pathogenicity of missense variants affecting the collagen IV α5 carboxy non-collagenous domain in X-linked Alport syndrome
Source: Sci Rep. 2022 Jul 4;12:11257. doi: 10.1038/s41598-022-14928-x (PMC9253329; doi:10.1038/s41598-022-14928-x)
Supplement: Supplementary file 1 — Supplementary Information. [file 41598_2022_14928_MOESM1_ESM.pdf]

Supplemental Material Table of Contents

CONSORTIUM NAME

Consortium members of Genomics England who did not directly contribute to this project are

[5]Ambrose, J. C. ; [5]Arumugam, P. ; [5]Baple, E. L. ; [5]Bleda, M. ; [5,6]Boardman-Pretty, F.; [5]Boissiere, J. M. ; [5]Boustred, C. R. ; [5]Brittain, H. ; [5,6]Caulfield, M. J.; [5]Chan, G. C. ; [5]Craig, C. E. H. ; [5]Daugherty, L. C. ; [5]de Burca, A. ; [5]Devereau, A. ; [5,6]Elgar, G. 1,2 ; [5]Foulger, R. E. ; [5]Fowler, T. ; [5]Furió-Tarí, P. ; [5]Giess A. ; [5]Hackett, J. M. ; [5]Halai, D. ; [5]Hamblin, A.; [5,6]Henderson, S.; [5]Holman, J. E. ; [5]Hubbard, T. J. P. ; [5,6]Ibáñez, K.; [5]Jackson, R. ; [5,6]Jones, L. J.; [5,6]Kasperaviciute, D.; [5]Kayikci, M. ; [5]Kousathanas, A. ; [5]Lahnstein, L.; [5]Lawson, K. ; [5]Leigh, S. E. A. ; [5]Leong, I. U. S. ; [5]Lopez, F. J. ; [5]Maleady-Crowe, F. ; [5]Mason, J. ; [5,6]McDonagh, E. M.; [5,6]Moutsianas, L.; [5,6]Mueller, M.; [5]Murugaesu, N.; [5,6]Need, A. C.; [5]Odhams, C. A.; [5]Orioli A.; [5,6]Patch, C.; [5]Perez-Gil, D.; [5]Pereira, M. B.; [5]Polychronopoulos, D.; [5]Pullinger, J. ; [5]Rahim, T.; [5]Rendon, A.; [5]Riesgo-Ferreiro, P. ; [5]Rogers, T. ; [5]Ryten, M. ; [5]Savage, K. ; [5]Sawant, K. ; [5]Scott, R. H.; [5]Siddiq, A. ;[5]Sieghart, A.; [5,6]Smedley, D. ; [5,6]Smith, K. R.; [5]Smith, S. C. ; [5,6]Sosinsky, A.; [5]Spooner, W. ; [5]Stevens, H. E.;[5]Stuckey, A.; [5]Sultana, R. ; [5]Tanguy M.; [5,6]Thomas, E. R. A.; [5]Thompson, S. R. ; [5]Tregidgo, C. ; [5,6]Tucci, A. ; [5]Walsh, E. ; [5]Watters, S. A. ; [5]Welland, M. J.; [5]Williams, E. ; [5,6]Witkowska, K.; [5,6] Wood, S. M.; [5]Zarowiecki, M.; [5]Genomics England, London, UK, [6]William Harvey Research Institute, Queen Mary University of London, London, EC1M 6BQ, UK.

Supplemental Table 1. NC1 domain locations within the six human collagen IV α-chains.

Supplemental Table 2. Splicing predictions for all exonic single-nucleotide substitutions located within 3 bases of a canonical splice site.

Supplemental Table 3. Categorisation of all possible unique collagen IV α5 NC1 missense variants.

Supplemental Figure 1. Variant inclusion flowchart for the theoretical cohort comprising all possible collagen IV α5 NC1 missense variants.

Supplemental Figure 2. Multiple sequence alignment of the six human collagen IV α-chains.

Supplemental Table 1. NC1 domain locations within the six human collagen IV α-chains.

Chain	Isoform	NC1 domain (residues)	NCBI transcript reference sequence	NCBI protein reference sequence
α1	1	1439 – 1669	NM_001845.6	NP_001836.3
α2	1	1484 – 1712	NM_001846.4	NP_001837.2
α3	1	1437 – 1670	NM_000091.5	NP_000082.2
α4	1	1458 – 1690	NM_000092.5	NP_000083.3
α5	2	1461 – 1691	NM_033380.3	NP_203699.1
α6	A	1462 – 1691	NM_001847.4	NP_001838.2

Supplemental Table 2. Splicing predictions for all exonic single-nucleotide substitutions located within 3 bases of a canonical splice site.

Gene	Nucleotide change	Expected protein change	Type	MES score (Mutant)	MES score (WT)	MES score change (%)	Predicted to affect splicing	Database
COL4A5	c.4528G>C	p.Gly1510Arg	Splice donor site	2.24	6.04	-62.91	Yes	ClinVar
COL4A5	c.4708T>C	p.Cys1570Arg	Splice acceptor site	9.72	10.91	-10.91	No	LOVD
COL4A5	c.4709G>T	p.Cys1570Phe	Splice acceptor site	10.19	10.91	-6.60	No	ClinVar
COL4A5	c.4709G>C	p.Cys1570Ser	Splice acceptor site	10.93	10.91	0.18	No	LOVD, ClinVar
COL4A5	c.4709G>A	p.Cys1570Tyr	Splice acceptor site	10.45	10.91	-4.22	No	LOVD
COL4A5	c.4819A>G	p.Met1607Val	Splice donor site	3.10	3.85	-19.48	Yes	LOVD
COL4A5	c.4823A>G	p.His1608Arg	Splice acceptor site	11.66	11.30	3.19	No	gnomAD
COL4A5	c.4993A>T	p.Ser1665Cys	Splice donor site	5.75	9.65	-40.41	Yes	LOVD

Grey rows indicate variants predicted to affect normal splicing. Known exonic splicing variants are not shown. MES, MaxEntScan; WT, Wild type.

Supplemental Table 3. Categorisation of all possible unique collagen IV α5 NC1 missense variants.

a) All variants

		Residue: Conserved[a]	Residue: Not conserved	Total
**Variant:**	Any structural damage	196	78	274
	No structural damage	404	638	1042
	Total	600	716	1316

p<0.001, OR (95% CI)=3.96 (2.94, 5.38)

b) Excluding Cys variants

		Residue: Conserved[a]	Residue: Not conserved	Total
**Variant:**	Any structural damage	124	78	202
	No structural damage	404	638	1042
	Total	528	716	1244

p<0.001, OR (95% CI)=2.51 (1.82, 3.47)

OR (95% CI), Odds ratio (95% confidence interval).

[a] Residues fully conserved in all six human collagen IV α-chains.

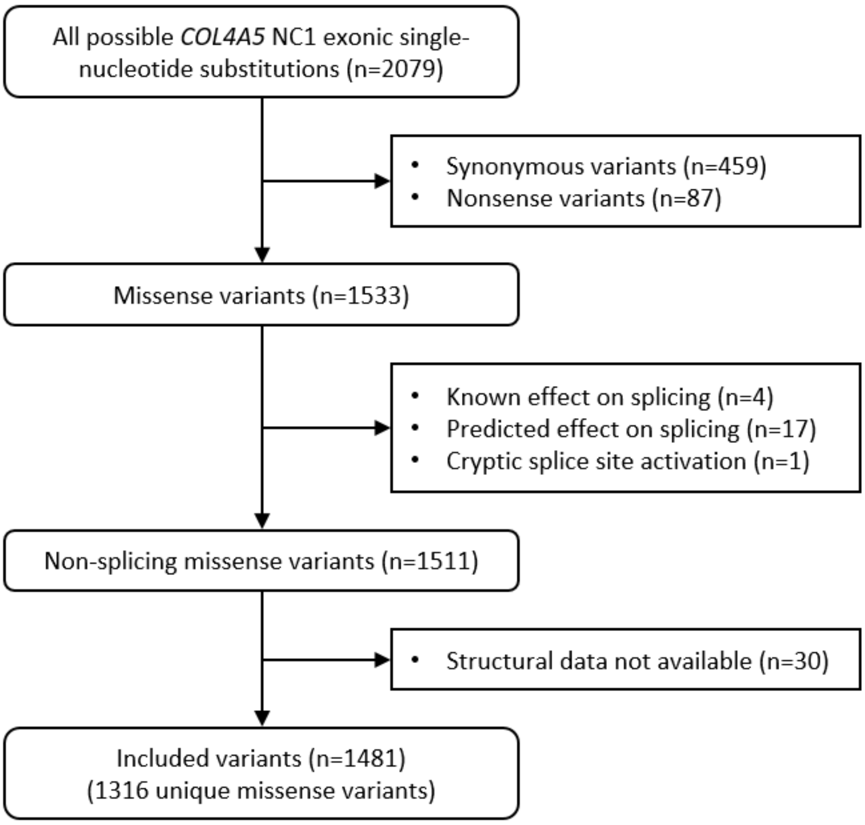

Supplemental Figure 1. Variant inclusion flowchart for the theoretical cohort comprising all possible collagen IV α5 NC1 missense variants. This was used to calculate the expected frequencies of structural changes for variants reported in LOVD.

```
α1      --TPSVDHGFLVTRHSQTIDDPQCPSGTKILYHGYSLLYVQGNERAHGQDLGTAGSCLRK     1496
α3      SPATWTTRGFVFTRHSQTTAIPSCPEGTVPLYSGFSFLFVQGNQRAHGQDLGTLGSCLQR     1496
α5      --TSSVAHGFLITRHSQTTDAPQCPQGTLQVYEGFSLLYVQGNKRAHGQDLGTAGSCLRR     1518
α2      ---RSVSIGYLLVKHSQTDQEPMCPVGMNKLWSGYSLLYFEGQEKAHNQDLGLAGSCLAR     1540
α4      -FGPGYLGGFLLVLHSQTDQEPTCPLGMPRLWTGYSLLYLEGQEKAHNQDLGLAGSCLPV     1516
α6      ---QSMRVGYTLVKHSQSEQVPPCPIGMSQLWVGYSLLFVEGQEKAHNQDLGFAGSCLPR     1518
                *: .. ***:   * ** *   :: *:*:*:.:*:::**.****  ****

α1      FSTMPFLFCNINNVCNFASRNDYSYWLSTPEPMPMSMAPITGENIRPFISRCAVCEAPAM     1556
α3      FTTMPFLFCNVNDVCNFASRNDYSYWLSTPALMPMNMAPITGRALEPYISRCTVCEGPAI     1556
α5      FSTMPFMFCNINNVCNFASRNDYSYWLSTPEPMPMSMQPLKGQSIQPFISRCAVCEAPAV     1578
α2      FSTMPFLYCNPGDVCYYASRNDKSYWLSTTAPLPM--MPVAEDEIKPYISRCSVCEAPAI     1598
α4      FSTLPFAYCNIHQVCHYAQRNDRSYWLASAAPLPM--MPLSEEAIRPYVSRCAVCEAPAQ     1574
α6      FSTMPFIYCNINEVCHYARRNDKSYWLSTTAPIPM--MPVSQTQIPQYISRCSVCEAPSQ     1576
        *:*:** :**  :** :* *** ****::   :**   *:    :  ::***:***.*:

α1      VMAVHSQTIQIPPCPSGWSSLWIGYSFVMHTSAGAEGSGQALASPGSCLEEFRSAPFIEC     1616
α3      AIAVHSQTTDIPPCPHGWISLWKGFSFIMFTSAGSEGTGQALASPGSCLEEFRASPFLEC     1616
α5      VIAVHSQTIQIPHCPQGWDSLWIGYSFMMHTSAGAEGSGQALASPGSCLEEFRSAPFIEC     1638
α2      AIAVHSQDVSIPHCPAGWRSLWIGYSFLMHTAAGDEGGGQSLVSPGSCLEDFRATPFIEC     1658
α4      AVAVHSQDQSIPPCPQTWRSLWIGYSFLMHTGAGDQGGGQALMSPGSCLEDFRAAPFLEC     1634
α6      AIAVHSQDITIPQCPLGWRSLWIGYSFLMHTAAGAEGGGQSLVSPGSCLEDFRATPFIEC     1636
        .:*****   ** **  * *** *:**:*.*.** :* **:* *******:**::**:**

α1      HG-RGTCNYYANAYSFWLATIERSEMF-KKPTPSTLKAGEL-RTHVSRCQVCMRRT-    1669
α3      HG-RGTCNYYSNSYSFWLASLNPERMF-RKPIPSTVKAGEL-EKIISRCQVCMKKRH    1670
α5      HG-RGTCNYYANSYSFWLATVDVSDMF-SKPQSETLKAGDL-RTRISRCQVCMKRT-    1691
α2      NGGRGTCHYYANKYSFWLTTIPEQ-SFQGSPSADTLKAGLI-RTHISRCQVCMKNL-    1712
α4      QGRQGTCHFFANKYSFWLTTVKADLQFSSAPAPDTLKESQAQRQKISRCQVCVKYS-    1690
α6      SGARGTCHYFANKYSFWLTTVEERQQFGELPVSETLKAGQL-HTRVSRCQVCMKSL-    1691
         * :***::::* *****:::     *   *  .*:* .   .  :******::
```

Supplemental Figure 2. Multiple sequence alignment of the six human collagen IV α-chains. Chains are grouped into α1-like chains (α1, α3, α5; above) and α2-like chains (α2, α4, α6; below). Positions with a single, fully conserved residue are indicated with an asterisk (*). Alignment was performed using Clustal Omega (https://www.ebi.ac.uk/Tools/msa/clustalo/).
